# Telephone-based communication training in the era of COVID-19

**DOI:** 10.3205/zma001416

**Published:** 2021-01-28

**Authors:** Clemens Ludwig, Dietrich Stoevesandt, Christiane Ludwig, Vivien Fritsche

**Affiliations:** 1 Martin Luther University Halle-Wittenberg, Dorothea Erxleben Lernzentrum, SkillsLab, Halle (Saale), Germany

**Keywords:** medical education, communication training, telephone communication, simulation person, patient simulation, COVID-19

## Abstract

**Introduction: **In the wake of the COVID-19 pandemic, alternatives to established and proven formats had to be found in university teaching within a very short time. In the case of the SkillsLab at the Dorothea Erxleben Lernzentrum Halle (Saale) at the Martin Luther University Halle (Saale)-Wittenberg, this meant in relation to the communication courses that a considerable proportion of the simulation participants of advanced age or with pre-existing conditions were suddenly no longer available for conversation simulations in teaching.

**Project description: **In the course of the seminar “Conversation with relatives – dealing with relatives” in the 8^th^ semester, the conversation simulation was therefore adapted at short notice and converted into a telephone conversation. Thus, the simulation subjects were able to participate remotely and the students had the opportunity to test their doctor-patient conversation skills with regard to telephone calls in a safe environment.

**Results: **The focus on nonverbal techniques and the departure from the usual face-to-face setting was perceived by students and simulation subjects alike as a positive stimulus and particularly challenging. The lack of visual impressions had made empathic conversation more difficult.

**Discussion and Conclusions: **The positive experiences from this project should be used to expand the communication curriculum in the future to include telephone-based conversations with simulation subjects. Ideally, it would then be possible for the simulation persons to be present in the future after the conversation for feedback mediation and group discussion.

## Zusammenfassung

**Einleitung:** Im Zuge der COVID-19 Pandemie mussten in der universitären Lehre in kürzester Zeit Alternativen zu etablierten und bewährten Formaten gefunden werden. Im Falle des SkillsLabs im Dorothea Erxleben Lernzentrum Halle (Saale) an der Martin-Luther-Universität Halle (Saale)-Wittenberg bedeutete dies bezogen auf die Kommunikationskurse, dass ein beachtlicher Teil der Simulationspersonen im höheren Alter oder mit Vorerkrankungen plötzlich nicht mehr für Gesprächssimulationen in der Lehre zur Verfügung stand.

**Projektbeschreibung: **Im Zuge des Seminares „Angehörigengespräch – Umgang mit Angehörigen“ im 8. Fachsemester wurde die Gesprächssimulation deshalb kurzfristig angepasst und in ein Telefonat umgewandelt. Somit wurde es den Simulationspersonen ermöglicht aus der Ferne mitzuarbeiten und die Studierenden hatten die Möglichkeit in sicherer Umgebung ihre Arzt-Patienten-Gesprächsführung in Bezug auf Telefonate zu erproben.

**Ergebnisse:** Der Fokus auf nonverbalen Techniken und das Verlassen des üblichen Face-to-Face Settings wurde gleichermaßen durch die Studierenden und die Simulationspersonen als ein positiver Impuls und besondere Herausforderung wahrgenommen. Das Fehlen der optischen Eindrücke habe eine empathische Gesprächsführung schwieriger gemacht.

**Diskussion und Schlussfolgerungen: **Die positiven Erfahrungen aus diesem Projekt sollen genutzt werden um das Kommunikationscurriculum zukünftig um telefongestützte Gespräche mit Simulationspersonen zu erweitern. Im Idealfall ist es dann möglich, dass die Simulationspersonen zukünftig nach dem Gespräch zur Feedbackvermittlung und zur Gruppendiskussion anwesend sein können.

## Introduction

In the wake of the COVID-19 pandemic, digital alternatives to previously established formats of university teaching suddenly had to be found. The hygiene concept of the medical faculty of the Martin-Luther-University Halle (Saale)-Wittenberg allowed the delivery of face-to-face courses starting in May 2020, as it was not possible to deliver the teaching units on practical and communication skills digitally while ensuring an adequate teaching experience. Meanwhile, exposure of elderly - or simulation persons (SP) [1] with pre-existing conditions in the context of the communication seminars should be avoided at all costs. Thus, it was necessary to establish an alternative to the previously used face-to-face setting in the short term. Mohos and colleagues [2] recommend video chat software such as Zoom to continue communication seminars online. However, we deliberately decided against it, as we could thus ensure that the teaching, as well as the employment of all participants, could be maintained largely independent of the private technical equipment (computer with webcam, Internet connection, software if necessary) of the SP. In addition, we were able to expand the content of the seminar to include the professionally relevant aspects (telephone as an acoustic business card [3]) and challenges (including limited impressions, susceptibility to interference [4]) of telephoning. 

## Project description

**Figure 1:**
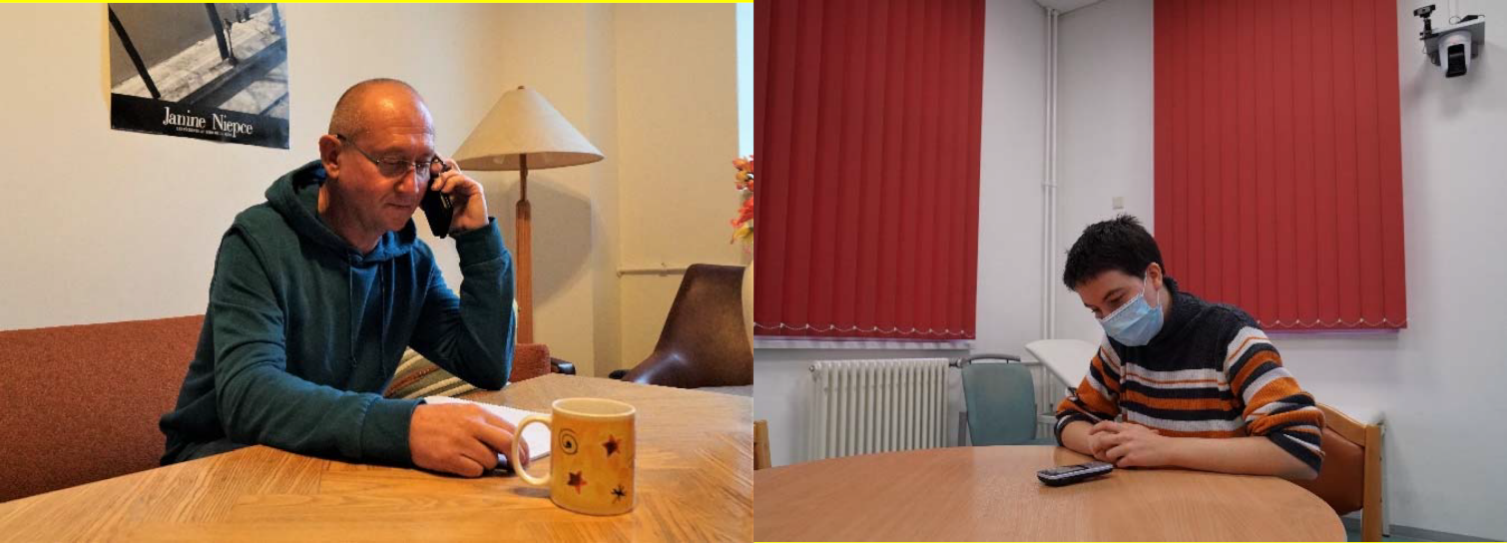
(left) A simulation patient in the role of the grandfather in the seminar “Talking to relatives – dealing with relatives” at home; (right) A student in the role of the assistant physician in the emergency department at DELH.

Communication and social skills are a basic qualification for medical staff [5]. In the SkillsLab of the Dorothea Erxleben Lernzentrum Halle (Saale) (DELH), students of human medicine undergo a longitudinally integrated communication curriculum during their studies, starting in the first semester. The courses are usually taught in a rotation principle based on small groups (max. 4-5 persons). To ensure the most believable performance possible, all communication seminars and simulations so far have been conducted on-site using SP to thus be able to include body language and facial expression in the performance in a standardized way [1]. The available SP pool includes student SPs (about 20-30 years old) as well as older SPs up to 80 years old. For some SPs, deployment was suddenly no longer readily possible. Therefore, in principle, other ways had to be found overnight to maintain teaching and examinations at a comparable quality. The SPs concerned signaled their willingness to work remotely. As part of the seminar “Talking to Relatives – Dealing with Relatives”, the students of the 8^th^ semester had the opportunity to conduct a conversation with a grandparent of a young child injured in a traffic accident. The concept, as well as the structure of the seminar could be continued unchanged and was supplemented by the peculiarities and characteristics of telephone calls: among other things, starting with the greeting with a clear mention of one’s own name and the function, respectively the intention of the conversation [3], [6], up to dealing with the missing mimic input. Misunderstandings, for example, cannot simply be read from a questioning facial expression. Students were then introduced to the case and provided with key medical information about the grandson’s condition. One person was designated to lead the conversation, and the remaining students moved to an observation room. Now the phone call took place in which the students introduced themselves as emergency room residents according to the scenario. Through the loudspeaker function it was also possible for the teacher and the observing students to follow the conversation. The SPs received a role script adapted to this scenario in advance. At the end of the call simulation, debriefing and reflection with the SP, the instructor, and the small group took place over the loudspeaker of the telephone (see figure 1 (Fig. 1)). 

## Results

In order to keep the risk of infection low, the usual pen & paper evaluation was not conducted during the summer 2020 semester, so the following points emerged mainly from debriefing with students. Students reported a greater “fear” of making a phone call. The reason for this would have been the feeling of not being able to adequately understand the interlocutor's condition in order to be able to react sufficiently well. The students noticed that it was more often necessary to give the SP space for follow-up questions after larger sections, or to summarize information or have it summarized [4]. The otherwise well usable nonverbal techniques (e.g.: facing posture, open gestures and facial expressions) did not add any value. Compared to a direct conversation, the benefit of the active listening techniques (e.g.: echoing, paraphrasing, and summarizing) became more apparent to guide the interlocutor through the conversation. Specifically, the lack of mimic input, longer pauses, or silence were experienced as irritating. Overall, the simulation variation was found to be enriching due to the inclusion and reappraisal of a basically familiar process such as telephoning. This short-term change also presented a new challenge for the SPs due to the described peculiarities of telephone calls. However, the quality of the feedback provided by the SPs was not diminished. Although the SPs did not have to refer to body language, they were now able to discreetly make concrete notes during the conversation in order to point out noteworthy phrases and to formulate even more specific needs. From the teachers' point of view, the changed concept could be integrated well. Due to the pragmatic implementation, the susceptibility to errors caused by technology or operating errors was minimized and thus did not create any additional burden. 

## Discussion and conclusion

The response by students and SPs to this short-term change was positive (professional relevance, communicative challenge, maintenance of teaching [3], [4], [6]). There was a desire to implement this in other seminars as well. We have carried these desires and experiences over into the 2020/2021 winter semester and are again offering a telephone-based communication station. With the change that healthy SPs are present in our premises and can join the group for debriefing and reflection. A closer look using standardized evaluation forms will give us deeper insights into the pros and cons of this new setting in the future. 

## Competing interests

The authors declare that they have no competing interests. 

## References

[1] Sommer M, Fritz A, Thrien C, Kursch A, Peters T (2019). Simulated patients in medical education - a survey on the current status in Germany, Austria and Switzerland. GMS J Med Educ.

[2] Mohos A, Mester L, Barabás K, Nagyvári P, Kelemen O (2020). Orvos-beteg kommunikációs gyakorlat szimulált pácienssel a koronavírus-járvány idején. (A COVID–19-pandémia orvosszakmai kérdései). Orv Hetil.

[3] Mazur HG (2009). Praxisführung: Das Telefon ist die akustische Visitenkarte. Dtsch Ärztebl.

[4] Holtel M, Enseleit I, Ewald W, Herbig N, Heun S, Neufang A, Pilz S, Pivernetz K, Rode S, Stapenhorst, K (2018). Arbeitshilfe bessere Kommunikation 07. Kommunikation am Telefon.

[5] Kurtz S, Draper J, Silverman J (2017). Teaching and Learning Communication Skills in Medicine.

[6] Tischler M (2020). COVID-19 - Durchbruch für Telemedizin, Homeoffice und digitale Anwendungen?. Dtsch Dermatol.

